# Comment on Kobroob et al. Effectiveness of N-Acetylcysteine in the Treatment of Renal Deterioration Caused by Long-Term Exposure to Bisphenol A. *Biomolecules* 2021, *11*, 655

**DOI:** 10.3390/biom11060888

**Published:** 2021-06-15

**Authors:** Liang-Jun Yan

**Affiliations:** Department of Pharmaceutical Sciences, College of Pharmacy, University of North Texas Health Science Center, Fort Worth, TX 76107, USA; liang-jun.yan@unthsc.edu; Tel.: +1-817-735-2386; Fax: +1-817-735-2603

Bisphenol A (BPA: 2,2-bis-(4-hydroxyphenyl)-propane) is an industrial chemical that is widely used in the production of epoxy resins and polycarbonate for food containers and plastic bottles [1]. This chemical is also known as an endocrine disruptor, as it is an environmental estrogen that can bind estrogen receptor and disrupt the estrogen signaling pathways [1]. At high temperatures or when BPA is not completely polymerized, this chemical can be liberated in drinking water and food, which can then be absorbed into the human body [2]. Indeed, exposure to BPA has been linked to incidences of type 2 diabetes, heart disease, and chronic kidney disease [1]. Moreover, epidemiological studies have demonstrated that there are increased levels of urinary albumin in individuals who exhibit high blood BPA [3,4], indicating that the kidneys are one of the organs that can sustain injury by persistent BPA exposure. In fact, the kidney deposited the highest amount of BPA among all the organs examined in a study using mouse BPA exposure as a model [5], demonstrating renal dysfunction that is correlated with elevated levels of plasma BPA. Nonetheless, despite numerous studies, the underlying pathology of BPA-induced kidney injury remains to be elucidated, and approaches that can be applied to fight BPA-induced chronic kidney disease (CKD) also remain to be explored.

Oxidative stress associated with mitochondrial dysfunction has been thought to be one of the main mechanisms underlying BPA-induced CKD [6,7,8]. Therefore, antioxidants targeting mitochondrial oxidative stress and abnormalities should provide novel insights into strategies that could be designed to counteract BPA-induced chronic kidney toxicity. A recent study by Kobroob A. et al. [9] in the journal *Biomolecules* demonstrated the applicability of a well-known antioxidant N-acetylcysteine (NAC) in restoring kidney function after BPA-induced injury. Importantly, the study focused on the mitochondrial mechanisms of BPA-induced CKD that can be corrected by NAC.

Specifically, the authors exposed rats to BPA (50 mg/kg/day) for a period of 16 weeks with proper vehicle controls. At week 12, BPA-treated rats were orally administered with NAC (100 mg/kg/day) for 4 weeks (Figure 1), also with proper vehicle controls. Rats were sacrificed at the end of week 16 and experimental samples, including blood, urine, and the kidneys, were collected for analysis of kidney function and measurements of mitochondrial function and oxidative stress. The authors’ findings are summarized in Figure 2. After BPA exposure, the authors found that BPA induced less body weight gain and a decrease in kidney weight. Also, BPA showed no effects on kidney organ index (kidney weight vs. body weight). However, BPA exposure led to increased glomerular atrophy, increased apoptosis, and increased mitochondrial fragmentation and deformity. These dysfunctional changes were concurrent with increased production of reactive oxygen species and reactive nitrogen species, increased lipid peroxidation, and decreased renal antioxidant capacity. Mechanistically, the authors demonstrated that BPA-induced chronic kidney toxicity was modulated by the AMPK-Sirt3-SOD2-axis and could be counteracted by NAC treatment. As outlined in Figure 2, all the impairments induced by BPA could be restored or corrected by NAC. This study thus further demonstrates the usefulness and efficacy of NAC in combating BPA-induced chronic kidney toxicity.

As is often the case for most studies, several questions remain to be addressed for this particular study on NAC treatment of BPA-induced kidney toxicity. First, the mitochondrial superoxide generation site(s) that may be modulated by BPA is not known. Second, mitochondrial protein targets showing selective oxidative damage upon BPA exposure and attenuation of such damage by NAC remain to be explored. Additionally, future studies are needed to evaluate whether there are any additive renal protective effects on BPA-induced chronic kidney toxicity when NAC is co-administered with other well-known antioxidants, such as lipoic acid [10,11]. Finally, as exposure to BPA is inevitable in human daily life and alternative chemicals to supplant BPA are not feasible yet, more studies elucidating the mechanisms of BPA-induced kidney toxicity remain to be conducted.

## Figures and Tables

**Figure 1:**
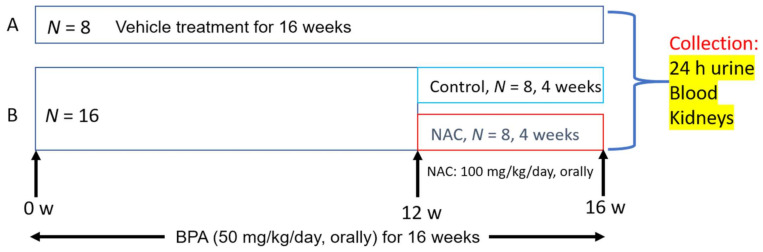
Experimental scheme and design of bisphenol A (BPA: 2,2-bis-(4-hydroxyphenyl)-propane) administration and N-acetylcysteine (NAC) treatment. Both BPA and NAC were administered orally.

**Figure 2:**
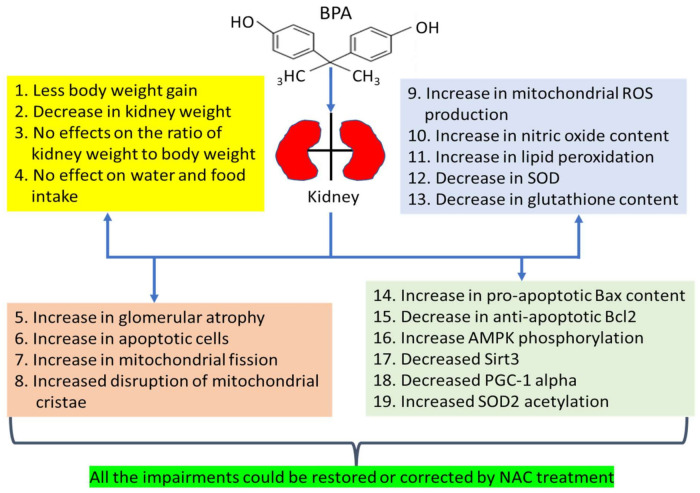
Various detrimental effects of BPA on the kidney and kidney mitochondria. All the impairments could be restored or corrected by 4 weeks of NAC treatment. ROS, reactive oxygen species; SOD, superoxide dismutase.
